# Controversies in the Treatment of Early Stage Endometrial Carcinoma

**DOI:** 10.1155/2012/578490

**Published:** 2012-03-26

**Authors:** Joshua Z. Press, Walter H. Gotlieb

**Affiliations:** Division of Gynaecologic Oncology, Segal Cancer Center, Jewish General Hospital, McGill University, Montreal, QC, Canada H3T 1E2

## Abstract

Despite the publication of numerous studies, including some multicentered randomized controlled trials, there continues to be vigorous debate regarding the optimal management of early stage endometrial cancer, including the extent of surgery and the role of adjuvant chemotherapy and radiation. Resolving these questions has become increasingly important in view of the increase of endometrial cancer, related to the aging population and the alarming incidence of obesity. Furthermore, there are more surgical challenges encountered when operating on elderly patients or on patients with increased BMI and the associated comorbidities, such as diabetes, hypertension, heart disease, and pulmonary dysfunction. This paper will focus on the advantages of minimally invasive surgery, the value of lymphadenectomy including sentinel lymph node mapping, and some of the current controversies surrounding adjuvant chemotherapy and radiation.

## 1. Introduction

The reader should be aware that most studies predate the present staging system (Table 1). Women with endometrial adenocarcinoma typically present with vaginal bleeding that prompts a pelvic examination and endometrial biopsy. The biopsy gives a general indication of the histology and grade. Endometrioid histology accounts for most cases (80%) and is graded from 1 to 3 depending on the degree to which normal architecture is lost and the extent of nuclear atypia. The remaining 20% of cases consist of nonendometrioid histology such as serous and clear cell histology, which are considered high grade because they are generally more aggressive and less predictable, with up to 50% lymph node metastases.

Unless patients are very poor surgical candidates, most physicians will initiate treatment with surgery. Typically the surgery includes hysterectomy and bilateral salpingoophorectomy, with or without lymphadenectomy, performed via traditional laparotomy, conventional laparoscopy, or robotic-assisted laparoscopy. Following surgery, the need for adjuvant treatment will be based on the final pathologic grade and histology, that can differ from the preoperative biopsy in up to 30% of the cases. The surgical specimen will in addition provide information about the depth of invasion, lymphovascular space invasion (LVSI), and tumor size, as well as the extent of extrauterine disease if lymphadenectomy or peritoneal biopsies are performed. The patient's risk of recurrence can then be estimated based on these pathologic factors (Table 2) leading to the risk-benefit analysis of adjuvant treatment, whether this adjuvant therapy will be in the form of radiation (vaginal brachytherapy or teletherapy), chemotherapy, or both [1]. A small proportion of patients are found to have advanced stage disease at presentation with pelvic/para-aortic lymph node or intra-abdominal metastases. These patients will require multimodal treatment with chemotherapy and radiation and tend to have a poor prognosis.

For early stage patients, developing the best management plan involves a fine balance between the risk of recurrence resulting from undertreatment and the risk of complications from overtreatment. Overtreatment can occur both from surgical interventions such as lymphadenectomy and adjuvant therapies such as radiation and chemotherapy. 

## 2. Surgical Approach: Traditional Laparotomy versus Conventional Laparoscopy versus Robotic-Assisted Laparoscopy

The traditional surgical technique for endometrial carcinoma has involved a laparotomy, either through a low transverse or a midline abdominal incision. With the increasing incidence of obesity and associated medical conditions this choice of surgical approach has come into question, in view of the prolonged recovery and increased wound complications. Following the first laparoscopic pelvic/para-aortic lymph node dissection for gynecologic cancer, reported by Querleu et al. in 1992 [2], there has been increasing utilization of the laparoscopic approach for endometrial cancer staging. This culminated in several recent randomized controlled trials comparing laparotomy to laparoscopy for surgical staging of endometrial carcinoma. The first study organized by the GOG (LAP2) randomized patients with clinical stage I-IIA endometrial cancer to hysterectomy with complete pelvic and para-aortic lymph node dissection by either laparoscopy or laparotomy [3]. The study enrolled 1696 patients to the laparoscopy group and 920 to the laparotomy group. Although the preliminary outcome data that showed similar disease-free and overall survival has just been presented as an oral communication at the SGO in 2010, complete information on surgical outcome data has already been published [4]. There was no significant difference in node positivity rate (9%), positive cytology rate, or detection of advanced stage disease (17%). The laparoscopy group was found to have an improved safety profile with fewer postoperative complications (*P* < 0.001), less antibiotic use (*P* < 0.001), and shorter hospital stay (*P* < 0.001). Patients in the laparoscopy group also had higher quality of life scores (*P* < 0.001), with better physical functioning, better body image, less pain (*P* < 0.001), earlier resumption of normal activities (*P* < 0.003), and earlier return to work over the 6 weeks following surgery. These differences remained up to 6 months after surgery (*P* < 0.04). Although this study demonstrated that laparoscopy was feasible and beneficial, the overall conversion rate from laparoscopy to laparotomy was as high as 26%, and this rate increased to 57% in women with a body mass index (BMI) greater than 40 kg/m^2^. Considering that women with endometrial carcinoma frequently have a BMI greater than 40, a significant proportion of patients would not benefit from this minimal invasive approach and end up with a laparotomy with the associated wound infection and breakdown. The second trial that randomized endometrial cancer patient to either laparotomy (*n* = 142) or laparoscopy (*n* = 190) was performed in Australia, New Zealand, and Hong Kong (LACE study) and also showed significantly shorter hospital stay and improved quality of life at both 4 weeks and 6 months [5]. Although this study had a lower conversion rate (3.6%), surgeons were permitted to omit lymphadenectomy depending on the BMI and medical fitness of patients, resulting in only 52% of patient having dissection of pelvic nodes, para-aortic nodes, or both. A third randomized trial performed in The Netherlands also confirmed that a laparoscopic approach provided benefit with respect to shorter hospital stay, less pain, and quicker resumption of daily activities, with a conversion rate in The laparoscopy group of 10.8% [6]. However, the influence of lymphadenectomy was not assessed in this study because lymphadenectomy is not a component of surgery for Stage I disease in the Netherlands.

A potential enhancement to the traditional laparoscopic technique has been provided by the creation of a robotic platform which facilitates minimally invasive procedures. The first report of robotic-assisted laparoscopic hysterectomy was published in 2002 [7]. Since that time the use of robotic-assisted laparoscopy for endometrial cancer staging has advanced rapidly, particularly in the United States. The switch from traditional laparoscopy to robotic-assisted laparoscopy has been criticized particulary due to the high cost of the robotic technology. Those who use the robotic platform argue that the extensive range of motion and three- dimensional visualization has simplified the performance of minimally invasive surgery, thereby minimizing the need for conversion to laparotomy. The largest published series of robotic-assisted laparoscopic endometrial cancer staging was reported in 2011 by Paley et al. [8]. The series compared 377 robotic surgeries for endometrial cancer to 131 laparotomies performed at the same institution. The major complication rate was significantly less with robotic surgery (20.6% versus 6.4%), particulary related to wound complications and infections. This difference in major complication rate became even more apparent in the cohort of patients with BMI > 40 (43.5% versus 11.3%). This data is consistent with the findings of the LAP2 study where minimally invasive technique was associated with fewer complications. However, in this robotic series the total conversion rate to laparotomy was 3.4%, with a conversion rate of only 5.8% in the 52 morbidly obese patients with a BMI greater than 40. In our first 100 endometrial cancer cases performed robotically, we had a 6% conversion rate, and all of these conversions were performed at the end of the robotic surgery by minilaparotomy to remove enlarged uteri that could not be delivered intact via the lower genital tract [9]. Technical improvements utilizing a large endobag introduced via the vagina at the end of the procedure allowed us to eliminate the conversions [10]. The major criticism of robotic-assisted laparoscopy is the cost of the robotic platform and instruments, which has become more of a concern with the rising costs of health care. Addressing the issue of cost requires complex comprehensive modeling that incorporates not only the surgical costs but also abstract variables such as the cost of postoperative complications and delays in return to work. There have been several studies modeling the cost of various surgical techniques for endometrial carcinoma. In the United States Bell et al. estimated the cost of endometrial cancer staging to be $12,943 by laparotomy, $7569 with traditional laparoscopy, and $8212 with robotic-assisted laparoscopy [11], and Barnett at al. estimated the cost to be $12,847 with laparotomy, $10,128 with traditional laparoscopy, and $11,476 with robotic-assisted laparoscopy [12]. In Canada, patients undergoing robotics had longer operating times (233 versus 206 minutes) but suffered fewer adverse events (13% versus 42%;*P* < 0.0001), lower estimated median blood loss (50 versus 200 mL, *P* < 0.0001), and median hospital stay was significantly shorter (1 versus 5 days; *P* < 0.0001). The overall direct and indirect hospital costs were significantly lower for robotics compared to the historical group (7644 versus 10368 CND; *P* < 0.0001) even when acquisition and maintenance cost were included (8370 versus 10368 CND; *P* = 0.0002). Within two years after surgery, the recurrence rate was lower in the robotic group compared to the historic cohort (*P* < 0.0001), although more mature data is eagerly expected [13]. When multiple cost variables are incorporated there appears to be a cost advantage to minimally invasive approaches.

## 3. Surgical Technique: Lymphadenectomy versus No Lymphadenectomy versus Sentinel Lymph Node Mapping

One of the most intense controversies in endometrial cancer revolves around the need for lymphadenectomy at the time of hysterectomy/BSO and the extent of lymphadenectomy that should be performed. A majority of cases present with clinical stage 1 disease (80%), without evidence of spread beyond the uterine cavity on either physical examination or radiologic imaging. However, the publication of GOG 33 in 1987 demonstrated that when complete surgical staging is performed, 22% of patients with clinical stage I disease have either lymph node metastases (12%), adnexal disease, intraperitoneal spread, or positive peritoneal cytology [14]. This study also identified important factors predicting lymph node involvement including tumor grade, depth of myometrial invasion, and lymphvascular space invasion. Since the publication of this study 3 strategies regarding lymphadenectomy have emerged. (1) Routine complete pelvic and para-aortic lymphadenectomy for all patients, with adjuvant treatment decisions based on the complete surgical staging. (2) Never perform routine lymphadenectomy, with risk of lymph node metastasis and recurrence being estimated based on uterine factors such grade, depth of invasion, LVSI, and tumor size. (3) Selective lymphadenectomy based on the intraoperative assessment of grade and depth of invasion. As will be seen in the following discussion, the subsequent studies of early-stage endometrial carcinoma have been influenced significantly by these different approaches to lymphadenectomy (Table 4).

### 3.1. Routine Lymphadenecomy

Following publication of GOG 33 there was a movement towards performing a systematic complete pelvic/para-aortic lymph node dissection in all patients with endometrial carcinoma. Potential benefits of this aggressive surgical approach include detailed information about the extent of spread, leading to improved understanding of prognosis, tailored adjuvant treatment, and possible therapeutic benefit. The prognostic value of lymph node status has been well defined, with 5-year recurrence free survival of 90% with negative nodes, 75% with positive pelvic nodes, and 40% with positive para-aortic nodes [15]. Those who support routine systematic lymphadenectomy believe that a negative lymphadenectomy allows for minimization of adjuvant treatment for patients with uterine confined disease. Furthermore, by identifying positive lymph nodes it is possible to define a group of patients who require even more aggressive treatment such as extended field radiation therapy to cover the para-aortic nodes and/or systemic chemotherapy. These potential benefits must be weighted against the unique intra/postoperative risks associated with lymphadenectomy including longer surgical time, risk of vascular/nerve injury, and lymphocyst/pelvic infection. Although there have been numerous retrospective studies indicating a therapeutic value to lymphadenectomy, several recent randomized trials discussed below have questioned this belief (Table 3).

### 3.2. No Lymphadenectomy

The least invasive lymph node strategy which has been studied more in European centers involves the complete omission of routine lymphadenectomy completely from the surgical procedure. Although, it should be noted that most study protocols using this strategy do recommend sampling of grossly suspicious lymph nodes. The risk of lymph node metastasis and recurrence is then estimated based on uterine risk factors such as grade, depth of invasion, LVSI, tumor size, and also patient age. Decisions regarding adjuvant treatment are then based on the degree of risk. Proponents of this strategy feel that this will limit the risks associated with lymphadenectomy, while still allowing for adequate treatment of patients with high risk of lymph node metastasis and high risk of recurrence. Critics point out that without pathologic assessment of lymph node status more patients will require adjuvant treatment, and the chosen adjuvant treatment may be inadequate if there are already lymphatic metastases.

The evaluation of the therapeutic value of systematic lymphadenectomy has been addressed by two recent multicentered randomized controlled trials which compared lymphadenectomy to no lymphadenectomy. Although both did not show any difference in survival after routine systematic lymphadenectomy, they have been the subject of extensive criticism [16, 17]. Benedetti-Panici et al. randomized 514 women less than age 75 with clinical stage I endometrial cancer to systematic pelvic lymphadenectomy or no lymphadenectomy [18]. Inclusion to the study required frozen section demonstrating myometrial invasion and stage IB-G1 were excluded. Para-aortic dissection was performed at the discretion of the surgeon, and bulky nodes (>1 cm) could be removed even in the control arm. The study found no significant difference in 5-year disease free survival (81% versus 82%) or 5-year overall survival (86% versus 90%) and no difference in site or pattern of recurrence. However, in this study the use of adjuvant treatment was left to the discretion of the treating physician, and significantly more patients in the no lymphadenectomy group received adjuvant radiation therapy (25.2% versus 16.7%). In contrast, more patients in the lymphadenectomy group received adjuvant chemotherapy. It should also be noted that removal of suspicious lymph nodes was a component of the no lymphadenectomy strategy, resulting in 16% of women in the no lymphadenectomy group having >6 nodes removed.

The second trial was the MRC ASTEC trial which randomized 1408 women with clinical stage I or II endometrial cancer to either systematic pelvic lymphadenectomy or no lymphadenectomy [19]. Para-aortic lymphadenectomy was left to the discretion of the surgeon, and removal of suspicious pelvic lymph nodes was permitted in the “no lymphadenectomy” group. Patients found to have intermediate- and high-risk disease (IAG3, IBG3, IC-all grades, IIA, serous/clear cell) were then randomized to external beam pelvic radiation therapy versus no external beam radiation as part of a combined trial with NCIC EN.5 which examined the utility of adjuvant external beam radiotherapy [20]. Patients found to have low-risk early-stage or advanced disease were treated according to physician preference. Vaginal brachytherapy was left to the discretion of the treating physician in all patients. The study found no difference in recurrence-free survival or overall survival between the routine lymphadenectomy and the “no lymphadenectomy” group. However, one must consider that the patients we expect to help most by performing routine lymphadenectomy are the 10% of intermediate-high risk patients who have occult pelvic and sometimes para-aortic lymph node metastases. This study included a large proportion of low-risk patients (50% low risk compared with only 37% intermediate and high risk) who would be unlikely to benefit from lymphadenectomy or adjuvant treatment. In addition, the study randomized intermediate-high risk patients to EBRT versus no EBRT, including those with positive pelvic lymph nodes (Stage IIIC). Although this group was small, the benefit of nodal dissection may have been limited because half of the patients who should have been triaged into adjuvant therapy were randomized to no additional treatment. The occult IIIC patients identified by lymphadenectomy are patients for whom we hope to find efficacious adjuvant treatment, possibly using systemic chemotherapy. In this study 50% of these patients received no adjuvant treatment, except possibly vaginal brachytherapy. Even among patients with advanced stage disease only 25% received adjuvant chemotherapy treatment. Although there was limited standardization regarding adjuvant treatment particularly in the low-risk and advanced stage disease, all patients were included in the survival analysis. Furthermore, the limited staging requirement of only pelvic lymph nodes neglects to identify patients who could benefit from extended field radiation treatment or chemotherapy due to positive para-aortic nodes. Lastly, despite randomization in the ASTEC study the lymphadenectomy group ended up with more high-risk patients, with 3% more poor histology, 3% more grade 3 lesions, 3% more +LVSI, and 10% more with deep myometrial invasion. Although each of these differences is small in magnitude, recall that the intermediate-high risk group for whom we expect adjuvant therapy to have the greatest influence makes up only 10–20% of patients with endometrial cancer. Thus any possible survival benefit to routine lymphadenectomy may have been obscured by the fact that the lymphadenectomy group were at higher risk to start with.

These 2 recent RCTs suggest that the removal of lymph node tissue may not have a large impact on survival. However, the design of these studies has not addressed the most important impact of lymphadenectomy which is to identify patients who can safely avoid adjuvant treatment and others who would benefit from more aggressive adjuvant treatment. In the Benedetti-Panici trial the “no lymphadenectomy” arm received significantly more adjuvant therapy than patients who had complete lymph node staging. Although comparing the true toxicity from lymphadenectomy and radiation therapy is difficult as both of these toxicities are likely underreported in literature, it is possible to extract data from some of the large randomized controlled trials. In the ASTEC study there were more adverse events in the lymphadectomy group versus no lymphadectomy with ileus (3% versus 1%), deep venous thromboembolism (1% versus 0.1%), lymphocyst (1% versus 0.3%) and major wound dehiscence (1% versus 0.3%). However, the extrapolation of these results is questionable in the current era of minimally invasive surgery, as only 6% of the ASTEC surgeries were performed laparoscopically. Furthermore, among the 94% performed by laparotomy a pfannenstiel incision was performed more frequently for patients without lymphadenectomy compared to with lymphadenectomy (49% versus 32%). These risks of lymphadenectomy must be compared with the potential complications of external beam radiation, which is required more often in patients in who lymphadenectomy is omitted. Radiation effects include both early and late complications such as enteritis, proctitis, cystitis, fistula formation, chronic gastrointestinal bleeding, hematuria, and diarrhea which can affect patients for their entire life [21]. Further discussion of these potential complications is presented below.

### 3.3. Selective Lymphadenectomy

Another strategy consists of performing a selective lymphadenectomy based on the intraoperative assessment of grade and depth of invasion. This is probably the strategy employed by many surgeons who are not devoted to one or the other approach, as it facilitates lymph node assessment in the patients at highest risk of lymph node metastasis, while minimizing operative morbidity. However, the utility of this strategy is limited by the inaccuracy of pre/intraoperative pathology assessment, where up to 15–25% of G1 biopsies are upgraded on final pathology [22]. Furthermore, gross intraoperative estimation of depth of invasion may be inaccurate especially for high-grade lesions, where small nest of tumor cells often invade deeply into the myometrium. Goff and Rice showed that for grade 3 lesions depth of invasion was only accurately determined in 31% of cases [23]. The specific threshold for proceeding with lymphadenectomy and the extent of lymphadenectomy (+/− para-aortic) remains operator dependent. An example of this strategy is the protocol developed by the Mayo clinic in which lymphadenectomy is omitted for patients with no invasion independent of grade, or for patients with G1/G2 tumors with myoinvasion <50% and primary tumor diameter <2 cm. For grade 3 tumors or for tumors invading more than 50% or larger than 2 cm an extensive dissection is performed with lymphadenectomy extending up to the renal vessels and excision of the gondal vessels at their insertion [24].

One of the drawbacks to the selective lymphadenectomy strategy is the risk of inaccurate intraoperative pathology assessment that would potentially benefit from pelvic/para-aortic lymph node dissection. The impact of this can now be reduced because a secondary operation to remove lymph nodes can be performed using a minimally invasive technique with minimal morbidity.

### 3.4. Sentinel Lymph Node Mapping

One elegant strategy that would resolve the controversy on lymphadenectomy is the implementation of sentinel lymph node mapping. This procedure is independent of the surgical approach, and can reduce complications associated with full lymphadenectomy, while providing information needed to plan adjuvant treatment. Several protocols have been examined using injection of blue dye, Technetium 99, or both into the cervix and/or uterine fundus [25]. Abu-Rustum et al. used an easy reproducible cervical injection to map the sentinel lymph nodes in grade 1 endometrial cancer and achieved intraoperative localization in 86% of patients [26]. In cases with successful localization, all node-positive cases were identified. A more recent French study (SENTI-ENDO) utilizing a similar injection protocol looked at 133 patients who underwent sentinel lymph node biopsies, followed by complete lymphadenectomy [27]. 90% of patients had at least one SLN detected, and of these 17% had pelvic lymph node metastases. There were 3 patients with false-negative SLN, but all were type II high-grade cancers with greater than 50% invasion. SLN upstaged 10% of low-risk and 15% of intermediate-risk patients. A recent meta-analysis found that pericervical injection had an increased detection rate compared to hysteroscopic or subserosal injection technique [28]. The combination of radiolabelled colloid and blue dye was associated with the highest detection rate and lowest false-negative rate. A national Canadian study on sentinel lymph node mapping in endometrial cancer, led by the GOC (Society of Gynecologic Oncology of Canada), is presently undergoing evaluation.

## 4. Role of Adjuvant External Beam Radiation Therapy in Intermediate High-Risk Early-Stage Disease

External beam pelvic radiotherapy (EBRT) has commonly been used as adjuvant treatment for high risk endometrial cancer. Recently, several large randomized controlled trials have assessed the benefit of adjuvant radiation treatment in early-stage patients with surgical-pathologic features indicating an intermediate- and/or high-risk of recurrence. The first of these studies, PORTEC-1 was a multi-institutional study in groups who did not perform routine lymphadenectomy [29]. In the PORTEC-1 study intermediate risk patients (G1 > 50% invasion, G2 any invasion, and G3 < 50% invasion) were randomized after hysterectomy without lymphadenectomy (except biopsy of suspicious nodes) to pelvic radiotherapy (4600 cGy) versus no further therapy. Stage IC-G3 patients were excluded, and vaginal brachytherapy was not given. Although there was a reduction in 5-year local recurrence rate (4% versus 14%, *P* < 0.001), there was no difference in 5-year overall survival (81% versus 85%, *P* = 0.37). A reanalysis of PORTEC-1 with 10 year overall survival still showed no difference (66% versus 73%, *P* = 0.09) [30]. Another feature of PORTEC-1 was that 31/35 (89%) of patients with vaginal recurrence had a complete response to salvage therapy with a 65% 5-year survival rate.

A similar study performed by the Gynecologic Oncology Group (GOG 99) randomized intermediate risk (IB, IC, IIA occult, IIB occult disease) completely staged patients (pelvic and para-aortic LN) to pelvic radiotherapy (5040 cGy) versus no further therapy [31]. As with PORTEC-1, although the 2-year locoregional recurrence rate was lower in the radiation group (3% versus 12%, *P* = 0.007), the 4-year overall survival was not significantly different (92% versus 86%, *P* = 0.557). The distant metastasis rate was similar (5% versus 6%), and 2/3 of the local recurrences could be salvaged with radiation therapy. In GOG99 there was a subgroup which performed very poorly without radiation treatment (older age, G2/G3, LVSI, and outer 3rd myometrial invasion). Both studies demonstrated that external beam radiation treatment could reduce the risk of local regional recurrence by about 2/3, but did not improve overall survival, regardless of whether routine lymphadenectomy was performed. Approximately 2/3 of patients with local regional recurrence could be salvaged with radiation treatment at time of recurrence. Thus overall local-regional control is similar regardless of adjuvant EBRT, it is just a question of whether the radiation treatment is given adjuvantly or after local recurrence. In both studies the radiation treatment did not seem to have any effect on the risk of distant recurrence (5-6%). Another component of useful data from these 2 studies is assessment of side effects from radiation treatment. In GOG99 there was significantly more grade 3/4 toxicity in the radiation arm (4.7% versus 0.5%), and 2 patients in the radiation arm died from radiation-induced gastrointestinal complications. In PORTEC 1 the toxicity was 25% in the radiation arm versus 6% in the control arm, with 3% of patients in the radiation arm experiencing grade 3/4 toxicity. A recent retrospective review suggests the development of radiation complications is accelerated after laparoscopic surgery (21 versus 45 months), but the incidence of grade 3 or 4 toxicity was higher after laparotomy (61.1 versus 14.3%) [21].

 Based on the major risk factors for recurrence in PORTEC-1 another study (PORTEC-2) was designed to compare EBRT with vaginal brachytherapy alone in a group high-intermediate risk patients. High-intermediate risk was defined as age > 60 with stage IC-G1/G2, stage IB-G3, or any age with Stage IIA, excluding IC-G3. Eligible patients were randomized to EBRT (4600 cGy) versus vaginal brachytherapy only [32]. There was no difference in vaginal recurrence rate (1.6% versus 1.8%, *P* = 0.74) or overall survival (79.6% versus 84.8%, *P* = 0.57). There was also no difference in the rate of locoregional relapse (vaginal or pelvic) or isolated pelvic recurrence. Vaginal brachytherapy alone was also associated with significantly less morbidity, with no added gastrointestinal toxicity [33]. The three studies showed that radiotherapy, whether EBRT or vaginal brachytherapy can influence locoregional recurrence but have little effect on distant recurrence or overall survival. This observation has shifted practice and research towards a systemic treatment focus.

## 5. The Role of Adjuvant Chemotherapy in Intermediate High-Risk Disease

Considering the lack of survival benefit from adjuvant external beam radiation therapy and lack of effect on distal recurrence rate, there has been increasing interest in incorporating adjuvant chemotherapy in the treatment of early-stage intermediate-high risk patients. Patients in PORTEC1 with stage IC-G3 disease had a five-year distant metastasis rate of 31%, and overall survival rate of 58% despite receiving EBRT. Although the impact of chemotherapy in the adjuvant setting is lacking, there are several studies demonstrating the effect of chemotherapy in advanced disease. The GOG performed a study (GOG122) for women with advanced stage endometrial carcinoma (Stage III/IV with postoperative residual disease) where patients were randomized to whole abdominal radiation versus chemotherapy (doxorubicin/cisplatin) [34]. This study showed significantly improved progression-free and overall survival with combination chemotherapy. Studies of other chemotherapy regimens in advanced endometrial cancer have resulted in the chemotherapy regimen being examined in the current GOG249 protocol for intermediate-high risk early-stage endometrial carcinoma: paclitaxel and carboplatin. GOG177 compared doxorubicin/cisplatin (AP) with a 3 drug regimen including taxol/doxorubicin/cisplatin (TAP) [35]. This study demonstrated improved response rate, progression-free survival and overall survival with the addition of paclitaxel but significantly increased toxicity with the 3-drug regimen. A current GOG study of chemotherapy in advanced endometrial cancer (GOG 209) is comparing the 3-drug regimen from GOG 177 (TAP) with a less toxic regimen (paclitaxel/carboplatin). The results of this study may significantly influence the regimen used for adjuvant treatment in early-stage high-risk disease. RTOG 9708 was a Phase II study for high-risk endometrial carcinoma in which 46 patients with G2/G3 lesions, greater than 50% invasion, cervical stromal invasion (IIB), or pelvic-confined extrauterine disease were treated with whole pelvic radiation with concurrent cisplatin chemotherapy sensitization, then followed by 4 cycles of taxol/cisplatin chemotherapy [36]. Although this was not a randomized controlled trail, the results are intruiging. In the stage IC, IIA, IIB patients there were no recurrences. More impressively in Stage III patients (*n* = 27) the 4-year disease-free survival was 72% and overall survival was 77%, which is significantly better than previous studies. The concept of combining radiation treatment with systemic chemotherapy as adjuvant treatment for intermediate-high risk early-stage disease is prevalent in both USA and European studies. As discussed the current GOG 249 study is randomizing women to either EBRT or a combination of vaginal brachytherapy and chemotherapy (Paclitaxel/Carboplatin). PORTEC III is also investigating this concept by randomizing high-risk Stage I,II, IIIA, IIIC patients to pelvic radiation alone versus pelvic radiation (with concurrent cisplatin) followed by carboplatin/paclitaxel chemotherapy.

## 6. Conclusions

Although there continues to be controversy regarding treatment of early-stage endometrial cancer, considerable progress has been made over the past several decades. Advancement in minimally invasive surgical techniques has allowed extensive staging procedures to be performed with significantly reduced patient morbidity. Although radiation therapy does not reduce overall survival, it does reduce the risk of locoregional recurrence. However, for most patients the use of vaginal brachytherapy alone can facilitate this localized benefit while minimizing long-term morbidity. Using surgical staging it is possible to avoid unnecessary adjuvant treatment in low-risk patients, while defining a group of higher risk early-stage patients who may benefit from more aggressive adjuvant therapy, such as systemic chemotherapy. Hopefully the result of currently enrolling randomized trials will further elucidate the most efficacious therapy for these high-risk patients.

## Figures and Tables

**Table 1:** Comparison of 1988 and 2010 surgical staging system for endometrial cancer.

Extent of tumor involvement	1988 staging	2010 staging
Endometrium only	1A	IA
Myometrium < 1/2	IB	IA
Myometrium > 1/2	IC	IB
Cervix mucosa	IIA	—
Cervix stroma	IIB	II
Uterine serosa/adnexa	IIIA	IIIA
Vagina/parametrial	IIIB	IIIB
Positive lymph nodes pelvic	IIIC	IIIC1
Positive lymph nodes periaortic	IIIC	IIIC2
Bladder or bowel mucosa	IVA	IVA
Distant metastases	IVB	IVB

**Table 2:** Risk of lymph node involvement and 5-year progression free survival based on depth of invasion and grade.

Depth of invasion	Grade	Risk of LN involvement	5-year PFS
Superficial (IA)	1 or 2	<3%	>95%
Superficial (IA)	3	~10%	~80%
Less than 50% (IB)	1 or 2		
Greater than 50% (1C)	1 or 2		
Greater than 50% (1C)	3	~30%	~50%
Cervical involvement (II)	any grade		

**Table 3:** Some important randomized trials comparing external beam radiation therapy to observation or vaginal brachytherapy alone.

	Treatment comparison	Local recurrence rate	5-year OS	Distant recurrence rate	G3/4 toxicity
PORTEC-1	EBRT versus observation	4% versus 14% (*P* < 0.001)	81% versus 85% (*P* = 0.31)	8% versus 7%	2% versus 0.002%
GOG99	EBRT versus observation	3% versus 12% (*P* = 0.007)	92% versus 86% (*P* = 0.6)	5% versus 6%	5% versus 0.5%
PORTEC-2	EBRT versus vaginal brachytherapy alone	2.1% versus 5.1% (*P* = 0.17)	80% versus 85% (*P* = 0.57)	6% versus 8%	2% versus <1%

EBRT—external beam radiation therapy, OS = overall survival.

**Table 4:** Some important studies of chemotherapy in endometrial cancer.

		PFS	OS	Toxicity issues
RCT : GOG 122 (advanced stage)	WAR versus chemotherapy (AP)	38% versus 50%	42% versus 55%	Treatment-related death 2% versus 4%
RCT : GOG 177 (Advanced stage)	Chemotherapy : AP versus TAP	5.3 versus 8.3	12.3 versus 15.3	Grade 3
		(months)	(months)	neuropathy
		(*P* < 0.01)	(*P* = 0.037)	1% versus 12%
Phase II : RTOG 9708 (All stages)	Chemoradiation : pelvic radiation (with concurrent cisplatin) + 4 cycles of TP	All stages: 81% Stage III = 72%	All stages: 85% Stage III = 77%	Grade 3 = 16% Grade 4 = 5%

RCT = randomized controlled trial, WAI = whole-abdominal radiation, AP = doxorubicin and cisplatin, TAP = paclitaxel, doxorubicin, cisplatin (+filgrastim), TP = paclitaxel and cisplatin, PFS = progression-free survival, and OS = overall survival.
